# Monitoring Water-Soil Dynamics and Tree Survival Using Soil Sensors under a Big Data Approach

**DOI:** 10.3390/s19214634

**Published:** 2019-10-24

**Authors:** Adrián Pascual, Rafael Rivera, Rodrigo Gómez, Susana Domínguez-Lerena

**Affiliations:** 1 School of Forest Sciences, University of Eastern Finland, P.O. Box 111, 80101 Joensuu, Finland; 2 Centro de Estudos Forestais, Instituto Superior de Agronomia, Universidade Técnica de Lisboa, Tapada da Ajuda, 1349-017 Lisboa, Portugal; 3 ICLAVES, S.L., 28003 Madrid, Spain; rafaelrivera@iclaves.es; 4 CESEFOR Foundation, Pol. Ind. Las Casas, 42005 Soria, Spain; rodrigo.gomez@cesefor.com; 5 SDL, Investigación y Divulgación del Medio Ambiente, 28491 Madrid, Spain; susanad@sdlmedioambiente.com

**Keywords:** remote sensing, urban forests, soil sensoring, precision forestry, proximal sensing, integrated systems

## Abstract

The high importance of green urban planning to ensure access to green areas requires modern and multi-source decision-support tools. The integration of remote sensing data and sensor developments can contribute to the improvement of decision-making in urban forestry. This study proposes a novel big data-based methodology that combines real-time information from soil sensors and climate data to monitor the establishment of a new urban forest in semi-arid conditions. Water-soil dynamics and their implication in tree survival were analyzed considering the application of different treatment restoration techniques oriented to facilitate the recovery of tree and shrub vegetation in the degraded area. The synchronized data-capturing scheme made it possible to evaluate hourly, daily, and seasonal changes in soil-water dynamics. The spatial variation of soil-water dynamics was captured by the sensors and it highly contributed to the explanation of the observed ground measurements on tree survival. The methodology showed how the efficiency of treatments varied depending on species selection and across the experimental design. The use of retainers for improving soil moisture content and adjusting tree-watering needs was, on average, the most successful restoration technique. The results and the applied calibration of the sensor technology highlighted the random behavior of water-soil dynamics despite the small-scale scope of the experiment. The results showed the potential of this methodology to assess watering needs and adjust watering resources to the vegetation status using real-time atmospheric and soil data.

## 1. Introduction

Increasing urban green area is a valuable adaptation policy to face climate change [1] and to mitigate its effects on human beings [2] while providing access to green areas for an increasing population [3]. In the urban areas of developed countries, the infrastructure and both agricultural and industrial activities guided fast and upscaling development during the last few decades, paving the way to an increasing urban sprawl that ignores the importance of green areas and urban forests during the still ongoing urban expansion. The recovery of vegetation within the urban sprawl is a very slow process [4] and, in many cases, human intervention is required to overcome both natural and human-related barriers [5]. Despite the complexity of the challenge, urban forests are regarded as feasible solutions for re-greening urban areas worldwide [6,7].

The recovery of forest cover in arid urban environments requires the implementation of technical solutions to create environmentally favorable conditions while optimizing resources [8]. In this regard, soil conditions play an important role in the framework of urban forestry, especially in Mediterranean semi-arid areas [9]. There is a clear relationship between soil properties and water-soil dynamics, which also explains water retention capacity and the flow of soil nutrients to plants, among other factors. The use of restoration techniques has been widely used in operational projects to improve tree survival in stressed and non-optimal conditions [10,11,12]. For instance, absorbent materials such as hydrogels contribute to a steady water infiltration [13], to stimulate biomass and tree growth [14], and to increase vegetation survival probability [15]. Similar effects can be achieved by using mycorrhizal inoculation, whose application in Mediterranean urban areas can overcome stress factors caused by soil conditions and drought episodes combined with high irradiance exposure [16,17]. Water-soil dynamics and the presence of microsite differences determine the effectiveness of restoration techniques [18]. Therefore, there is a need to capture the spatial pattern of water-soil dynamics for a better application of engineering solutions [19]. Disciplines such as agronomy or geology have already integrated spatially-explicit sensor-based solutions into experimental designs and operational projects [20,21]. Unfortunately, their application to address challenges in green urban planning remains somehow unexplored.

The increasing urban sprawl requires the integration of remote sensing-based platforms capable of combining multiple data sources oriented to support decision-makers while providing real-time information. The conversion of degraded land plots into new green urban areas requires effective restoration techniques and the monitoring of the accelerating process in the recovery of vegetation. In this research, we implemented a methodology to monitor water-soil dynamics and watering needs based on remotely-sensed soil and atmospheric data. With the aim of maximizing the presented sensor-based data-capturing scheme, we compared the influence of seasonal variation of rainfall runoff and water infiltration considering microclimate conditions on the establishment of a new urban forest. The research also evaluated how efficient alternative restoration techniques are improving the survival rate of five tree species in the study area, which has the following challenging conditions for establishing a new urban forest: (i) abandoned arid area within an urban environment, (ii) lack of watering infrastructure, and (iii) presence of severe drought episodes in a continental Mediterranean area.

The main objectives of our study were to (i) demonstrate how a methodology based on soil sensors, atmospheric data, and ground surveys can be implemented to monitor the establishment of a new urban forest and (ii) test the efficiency of the restoration techniques at improving the survival rate of the tree and shrub species selected. The presented wireless soil-atmospheric sensor-based design is a potentially valuable method to be further utilized in land-use planning and urban forestry.

## 2. Materials and Methods

### 2.1. Study Area

The study site is located within the urban sprawl of the region capital, Valladolid (Castilla y León, Spain). The urban forest project was located on the edge of a riparian zone and adjacent to an abandoned industrial facility and agricultural areas. The climate in the area is continental Mediterranean, with substantial intra-day variation of temperatures. Winters are cold (average monthly temperatures under 5 °C) with frequent fogs and frosts (61 days on average) and 8 days of snow per year. Summers are hot and dry, with maximums above 30 °C and daily minimal values slightly exceeding 13 °C. The average annual rainfall is 422 mm, uniformly distributed throughout the year except for July and August.

The experimental design was supported by local authorities by means of a new urban forest project. The aim of the project was to recover vegetation in an arid environment by minimizing infrastructure costs and watering needs and to be a showcase scenario from which to develop efficient green urban planning cases. The area has been abandoned for more than a decade and, as a result, there was a large amount of irregularly distributed coarse elements throughout the study site leading to site differences in terms of soil preparation and stone, which affect water-soil dynamics. The degraded conditions in the plot were severe enough that planting operations were regarded as unfeasible, but the idea of the experiment was to test the presented methodology under very challenging conditions (industrial arid environments in urban cities). Land-use maps and field inspections were used to evaluate the weights of coarse elements and their spatial variation; soil properties were analyzed by three trenches (soil samplings). The samples were collected at three different depths (lower limits 20, 50, and 120 cm respectively). The analysis of the soil showed poorly-developed soil layers with recent alluvial materials in depositional profiles and varying depth. Sandy loam and sandy soil were the soil textures in the study area (Table A1).

### 2.2. Experimental Design

**Figure 1:**
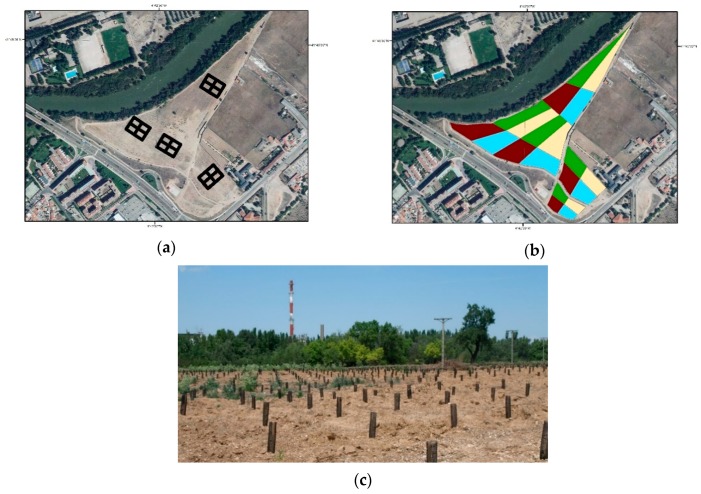
(**a**) Overview of the study area showing the four sampling plots; (**b**) Layout of study area showing the tested treatments: *NoTreat* (green), *Retainer* (light yellow); *Mycorrhiza* (dark red), and *Mixed* (blue); (**c**) Picture of the research experiment after the planting and the installation of the soil-sensoring network.

The extension of the new urban forest comprised 7.8 ha. Ground operation regarding planting, application of water retainers, and inoculation took place in May and June 2014. The spatial arrangement of the experimental design consisted of four randomly-distributed sampling plots. A sampling plot was defined as a sum of four blocks (Figure 1). On each block, four alternative planting techniques were tested: (i) plants with mycorrhizas inoculated during planting operations and in controlled conditions in the nursery (*Mycorrhiza*), (ii) plants in containers with a super-absorbent hydrogel solution when planting (*Retainer*), (iii) the combination of both *Mycorrhiza* and *Retainer* (*Mixed*), and (iv) bare-root planting (*NoTreat*), which was used as the benchmark that the three restoration techniques were compared against. The experiment consisted on 1,440 observations (i.e., planted trees). The design followed a hierarchical approach consisting of four sampling plots and 16 blocks on which the afore-said possibilities were tested. The area of the blocks ranged from 0.155 to 0.545 ha. In addition, the spatial arrangement of individuals within each block avoided consecutive repetitions of the same species in both the same column and row.

### 2.3. Selection of the Urban Forest Components

Recommendations from the municipal technicians and regional managers from the Forest Service of Castilla y León on species selection were followed. The following five species were finally selected: *Quercus ilex* L. subsp. *ballota* [Desf.] Samp. (*Qi*), *Pinus pinea* L. (*Pp*), *Juniperus thurifera* L. (*Jt*), *Prunus dulcis* (Mill.) D.A. Webb (*Al*) and *Acer campestre* L. (*Ac*). We aimed to test the response to treatments of autochthonous well-adapted and not as well-adapted species to the study area conditions. For instance, the use of *Quercus* spp. frequently presents developmental problems in reforestation (high first-stage mortality and development delay) compared to other species such as *Pp* or *Al*, which are highly recommended in reforestation programs in the area. The use of *Jt* is regarded as a challenge in the area due to its limited availability from local nurseries. Its use in reforestation projects is being promoted as it increases the ecological value of reforested areas [22]. The inclusion of Ac in the experiment was due to its widespread use as an ornamental tree, despite the species being more prone to sites with more pluviometry and shade. The restoration success of Ac in the study area conditions was regarded as an ambitious goal and its performance was expected to be a good indicator of the level of improvement provided by the proposed restoration techniques.

The candidate relationships between mycorrhiza species and tree species (i.e., study species) used expert-based knowledge and previous studies [23,24]. For the case of ectomycorrhizas, the prepared solution ranged from 5 × 10^5^ to 5 × 10^6^ spores/mL while for the Rhizophagus spp., a standard inoculation procedure was followed [25]. All inoculations were carried out in the field using diluted samples of 10 mL to inoculate each plant (Table A2). The effectiveness of water retainers in general and hydrogels in particular has been reported in similar studies [26]. The straightforward application of hydrogels in field labor operations and their good performance at a low cost were the reasons for their inclusion in the experiment. The research team selected the Stockosorb^®^ product based on previous experiences from the authors and previous studies with the product [27]. This water-absorbent polymer can retain up to 250 L of water using 1 kg of product, acting as a water reservoir in drought periods and gradually releasing water. As a result, soil volumetric water content increases. During our planting operations, about 150–250 mL of homogeneous product was used for the planting holes of approximately 10 L each.

### 2.4. Multi-Source Data Processing

**Figure 2:**
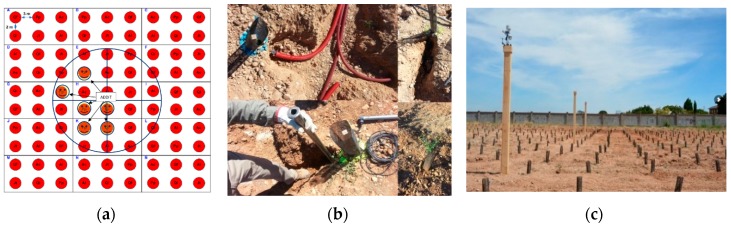
(**a**) Control and sensoring system showing the sensed plants and their connections using the ADDIT hub; (**b**) Ground operations during the installation phase; (**c**) Final layout of one of the sampling plots showing the local climate station.

A network of soil sensors was installed in each of the four sampling plots to monitor soil temperature and soil moisture every 30 min at two depths (20 and 40 cm). Capacitance-based soil moisture and soil temperature monitoring sensors were used in our study [19]. The soil sensors measured the frequency units of the capacitance (SUF) circuit generated by the electrodes of a probe. The scale frequency was converted to volumetric water content in the soil, expressed as a percentage. This conversion required a calibration process that depended on soil properties, among other factors [20]. The calibration process is detailed explained in Appendix B. To collect the information, we used *ADDIT S4* data loggers connected to a hub of communications that collected and sent data to a local micro-meteorological station placed within the study area. The station also collected independent data. Overall, 64 sensors were installed surrounding the center of each sampling plot (Figure 2). The correlation between sensed information and climatic variables needed to be accounted for when interpreting sensor measurements. For that purpose, a micro-climate station was installed in the center of the experiment. The station recorded the following variables of interest: temperature (°C), precipitation (mm), relative humidity (RH, %) and wind speed (Km h^−1^). In this way, sensor measurements could be associated in real time to the observed conditions.

**Figure 3:**
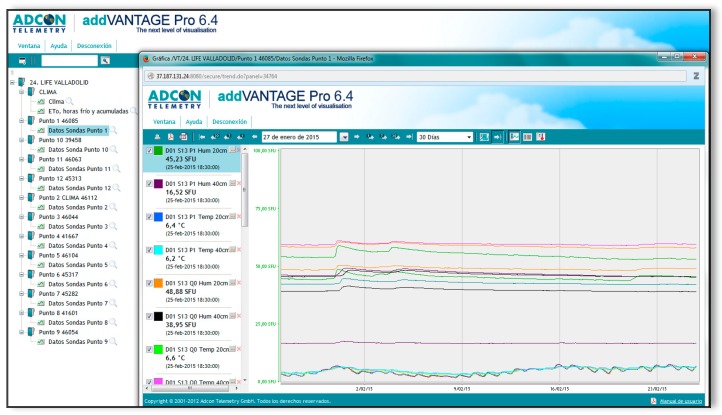
Example of the web-based data-fusion system in which records from the soil sensors and from atmospheric observations are synchronized every 30 min.

All measurements, soil, and atmospheric variables were synchronized at thirty-minute intervals and loaded to the server (Figure 3). Once the records were retrieved from the server system, the data was processed to compute one observation for each sensor every 30 min (Figure 3). Due to the small intra-day variation of the data and the magnitude of the set of sensor measurements, we also computed mean values per day. Therefore, we defined two datasets, one including all the observations and another including daily average observations. The first was used to monitor moisture content 24 h after each rainfall episode registered at the climate station, while the second was used for calculating the effect of treatments on the soil temperature and soil volumetric water content. The availability of a huge amount of data coming from the sensor network was an important feature of the experiment. In the following sections, we describe in detail the methodology used for gathering and analyzing data, as well as the theoretical and the empirical models that allowed us to obtain relevant conclusions on the real effect of the restoration techniques tested.

### 2.5. Evaluation and Monitoring of the Study

The experimental design consisted of 1440 individuals (360 per sampling plot). After the planting operations, four individuals were left out from the experiment (inadequate health status after transportation from the nursery). Therefore, 1436 observations were made since the planting was established in May 2014. Periodical inspections in the fields were carried out to evaluate the status of individuals and to compute the survival rate. The census was repeated every three months during the two first years after the establishment of the urban forest project. Field observations were used to feed survival models to estimate the probability of one tree surviving. The probability was computed individually for each tree and then the results computed bottom-up using the hierarchical design of the experiment (tree-level, block, sampling plot, and total). Survival rate was also computed for factors *Treatment* and *Species*. The auxiliary information provided by soil sensors was computed for 64 sensed individuals. The detected values of soil moisture and soil temperature were computed for each block within each sampling plot (an average of four sensed individuals per block). In this way, the detected (sensors) and measured information (field observations) matched at block level. The atmospheric variables were used to monitor the pluviometry along the two-year experiment with the aim of testing the effect of treatments after rainfall episodes (detected and recorded by the climate station installed in the study area). In the case of *Mycorrhiza* treatment, a control test was carried out on May 2015 by an independent laboratory aiming to compute the inoculation rate in the field after one year.

### 2.6. Statistical Analyses

#### 2.6.1. Kaplan-Meier and Cox Survival Models

Our main interest was to evaluate the effect of treatments on the survival chance of individuals. The survival probability was computed for each individual every time a new field observation was available. For this purpose, we applied the approach presented by Cox in [28], which accounts for the change in individual survival probabilities (Equation (1)) using an array of factors (Equation (2)). In this study, we accounted for the influence of treatments and species.

(1) $$h\left(t;e,r,x\right)=\underset{c\to 0}{\lim\;}\frac{P(t\leq T<t+c\;\vdots \;T\geq t,e,r,x)}{c},$$

(2) $$h\;\left(t;e,r,x\right)=k\left(e,r,x\right).h_{0}\left(t\right)$$

where *h* is the hazard function, *T* is the number of days between the start of the experiment and the time the given individual dies, *t* is the given day for which the probability is computed, *e* is the species, *r* is the tested restoration technique, $h_{0}\left(t\right)$ accounts for the time period, and *x* refers to other possible control variables such as the hierarchy levels in the experimental design (i.e., block). The computed survival rate in each of the surveys was graphically assessed based on the non-parametric model suggested in [29].

#### 2.6.2. Monitoring the Effect of Rainfall Episodes

The analysis of moisture retention was performed each month. We considered the hourly rate of rainfall (Rainfall) in each period as the dependent variable. The intensity of all rainfalls was higher than 10 mm per hour. The values of soil moisture content at the two depths (20 and 40 cm, respectively) were used as independent variables. The interaction between treatments (comparing each treatment with *NoTreat*) and the influence of the moisture content in the previous period was also acknowledged in the model, for which ordinary least squares (OLS) regression was used. As a showcase, we present the model formulation for the 20 cm depth case:

(3) $$Dif_{Hum_{20_{n}}}=\;\beta_{0n}+\;\beta_{1n}Treatment+\;\beta_{2n}Rainfall_{n}+\;\beta_{3n}Treatment*\;Rainfall_{n}\;+{Β}_{4n}Hum_{20_{n}}+\;\beta_{5}Rainfall_{}+\;\beta_{6n_{-1}}Rainfall_{n_{-1}}+\;\varepsilon$$

where *n* corresponds to each month of the 1:24 interval and *Dif_Hum_* is the difference between soil moisture content at the two depths (20 and 40 cm, respectively) in period *n*. *Rainfall* (the hourly rate of rainfall) for the given month (*n*) and the previous month (*n*−1) were included to evaluate the effect of the treatments, considering the initial conditions in month *n*, which was evaluated up to 24 (two-year experiment). The term *ε* is the independent and identically distributed error term with mean = 0 and assumed non-constant variance. We were mainly interested in the *β*_3*n*_ parameter to assess the efficiency of treatments.

#### 2.6.3. Moisture Retention and Soil Temperature

Using the daily observations dataset, we also estimated the soil volumetric content and temperature at 20 and 40 cm depths. The response variables *Tem*_20_, *Tem*_40_, *Hum*_20_, and *Hum*_40_ were estimated using the mixed-effects models [30] due to data hierarchy in our design. The levels were individual (*IdTree*), sampling plot (*Plot*), species (*Specie*), and treatment (*Treatment*). We used the *NoTreat* case and Plot #1 as reference levels when fitting the model. This way, model coefficients showed the performance of treatments compared to a reference scenario [31]. The models were fitted both in the original and logarithmic scales, altering the inclusion of factors in the fixed part. The statistical analyses were performed using the R statistical software [32] and the software packages “ggplot2” [33], “lme4” [30], and “nlme” [34]. The selected models were as follows:

1. *ME*_1_: A mixed-effect model including *Treatment* (our parameter of interest) in the fixed part and the rest of the factors and several possible combinations as random effects:
  fixed = “*Treatment*” (4)
  random = c((1|*IdTree*), (1 + *Treatment*|*Specie*), (1 + *Treatment*|*Plot*), (1|*Month*)) (5)
2. *ME*_2_: Factors *Plot* and *Specie* were added to the fixed part to highlight microsite differences in our experimental design and assess the effect of species in the limited time interval since the planting. Factors *IdTree* and *Month* were included in the random-effects part as follows:
  fixed = “*Treatment* + *Plot* + *Specie*” (6)
  random = c(1|*IdTree*, 1|*Month*) (7)

## 3. Results

### 3.1. Survival Analysis

**Figure 4:**
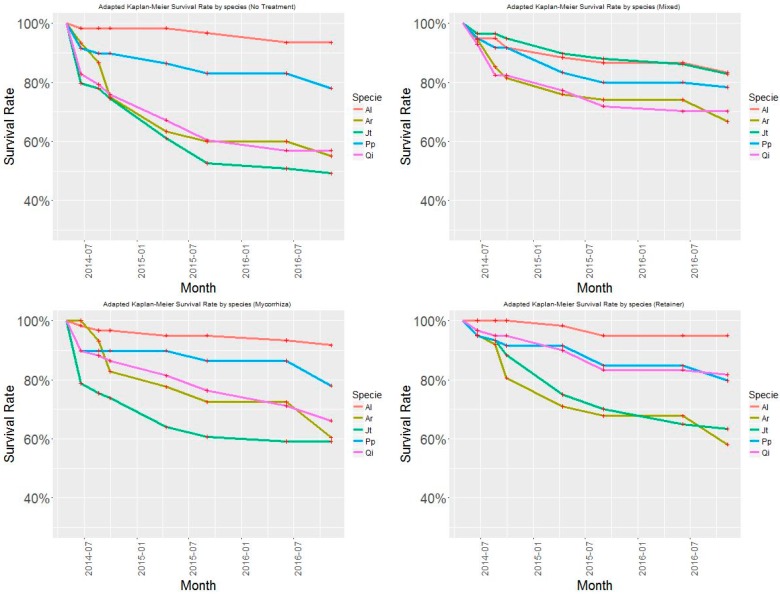
Evolution of survival rate by species and by treatment along the experiment lifecycle. The species are *Quercus ilex* (*Qi*), *Pinus pinea* (*Pp*), *Juniperus thurifera* (*Jt*), *Prunus dulcis* (*Al*), and *Acer campestre* (*Ac*).

At the end of the experiment, the computed survival rates at study level were 69% for *Mixed*, 60% for *Mycorrhiza*, 58% for *Retainer*, and 50% for the reference case *NoTreat*. In terms of species performance, the computed values were 91% for *Al*, 78% for *Pp*, 68% for *Qi*, 64% for *Jt*, and 60% for *Ac*. When the survival rates were computed by treatment, the improving effect of treatments was remarkable for *Jt*, and less so for both *Ac* and *Qi* (Figure 4). The behavior of the species *Pp* and *Al* remained uniform with and without the use of treatments. In all cases, early-stage tree mortality (boosted by extreme heat episodes during the first summer) was observed in all cases.

Considering all the species in the Cox model, the tested treatments increased survival, particularly for *Mixed* and *Retainer* cases that increased survival chances by 32% and 34% respectively. The observed effect was lower (17%) when using only mycorrhizas. According to the significance of the Cox model coefficients, treatments were highly significant (*p* value < 0.01) for *Mixed* treatment when using all observations and for *Jt* (increasing survival probability of 74%). The results for *Retainer* were highly significant for all observations together, specifically for *Qi* (64%), and less significant (*p* value < 0.1) for *Jt*. For the rest of the tested species, no statistical significance was observed for well adapted species *Pp*, *Al* and the more demanding *Ac*.

**Figure 5:**
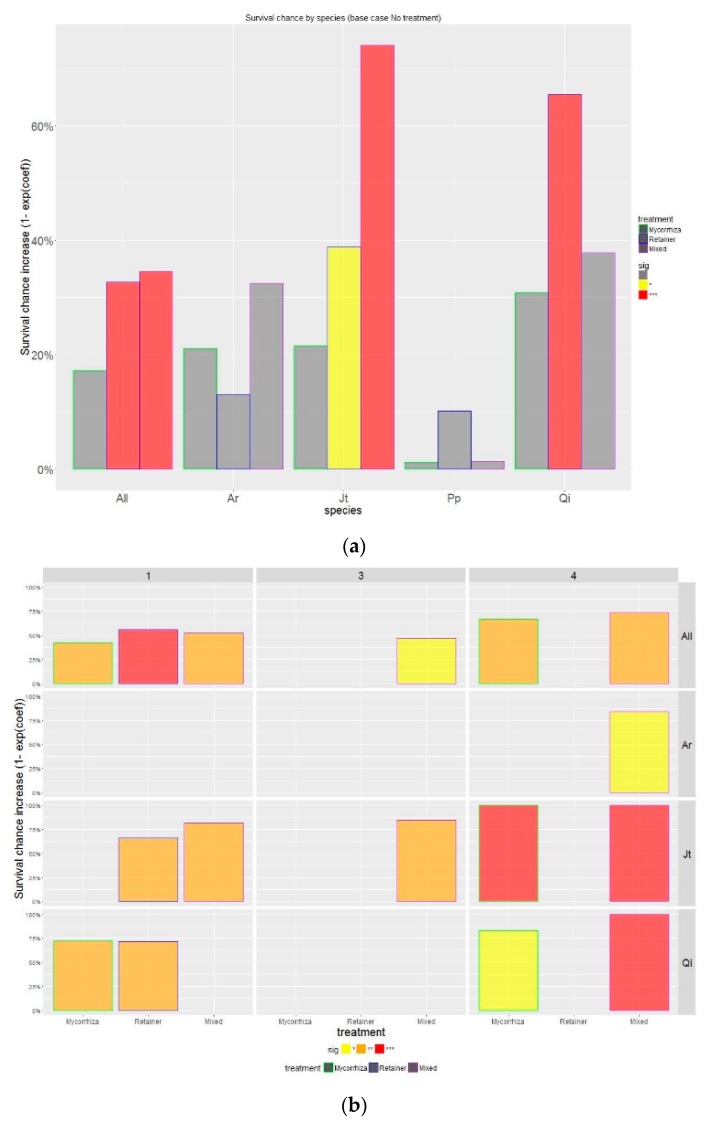
(**a**) Effect of treatments, displayed by species, on increasing survival chances. The significance of Cox model coefficients was highlighted in the graph using two levels of significance (* *p* = 0.1 (yellow); *** *p* = 0.01 (red); and non-significant cases (grey)). The species are *Quercus ilex*. subsp. *ballota* (*Qi*), *Pinus pinea* (*Pp*), *Juniperus thurifera* (*Jt*), *Prunus dulcis* (*Al*), and *Acer campestre* (*Ac*). Three bars are presented from left to right for each species corresponding to treatments *Mycorrhiza*, *Retainer* and *Mixed*, respectively; (**b**) Results presented by sampling plot using three levels of significance (* *p* = 0.1 (yellow); ** *p* = 0.05 (orange); *** *p* = 0.01 (red). Non-significant cases are presented in the background color. Plots #2 and #3 were combined for presentation due to the similar behavior on soil-water dynamics observed in the soil sensor datasets and during the calibration phase.

**Figure 6:**
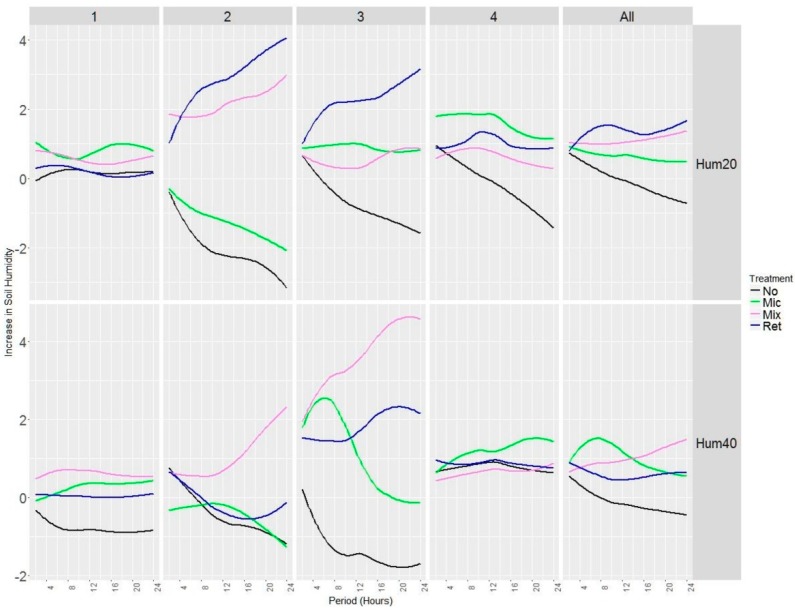
Effect of treatments on soil volumetric content after the 24 h since the last rainfall episode was registered. The *y*-axis represents the absolute increment of soil moisture content. The results at 20 and 40 cm depth are shown for each sampling plot and at the experimental design level (All).

The results were computed at plot level to assess the spatial variation of survival rate across the sampling design and evaluate the influence of soil properties. Based on the similar behavior of *Plot* #2 and *Plot* #3 at retaining rainfall at a 20 cm depth, their similar soil properties, and similar results in the tests, *Plot* #2 and *Plot* #3 datasets were evaluated as one (labelled as *Plot* #3 in Figure 6). For further correlation with soil moisture results, we presented the survival probability analysis for species *Ac*, *Jt*, *Qi* and all individuals combined (Figure 5) for each sampling plot.

### 3.2. Analysis of Soil’s Moisture Absorption of Rainfall

The positive effect of treatments to increase rainfall retention was assessed at two depths (Figure 6). The increment in moisture content at the 20 cm depth due to rainfall retention started to be significant (*p* value < 0.05) in all cases after 12 h from the rainfall episode. For the case of *Retainer*, the effect was already significant after 6 h. The effect of treatments was substantially lower at the 40 cm depth: model coefficients were only significant for the *Mixed* treatment and just after 18 h. We computed the difference (change) in the dependent variables (moisture at 20 cm and 40 cm) depending on the treatments for a rainfall of 10 mm in the *n* month period after a registered rainfall. Due to the complexity of the model, a graphical representation of the results can both better assess the effect of treatments and show the alternative infiltration patterns across the experimental design (Figure 6). The positive increment in moisture lasted longer and progressively increased for *Mixed* and *Retainer*, while the opposite behavior was observed in the *Mycorrhiza* treatment. When the results were downscaled and spatially displayed (i.e., for each sampling plot), we observed substantial variation in rainfall retention and in the way it progressed. The use of retainers was the most effective alternative to increase soil moisture content at the 20 cm depth in all plots while the *Mixed* treatment was the most significant at the 40 cm depth for *Plot* #2 and *Plot* #3.

### 3.3. Effect of Treatments on Soil Moisture and Temperature

Model coefficients showed the minor contribution of treatments to increase moisture content at the 20 cm depth. The results were not significant except for the *Mycorrhiza* treatment (*p* value < 0.1) in the *ME*_1_ model (Table 1). When factors *Specie* and *Plot* were added to the fixed part of the model, the results showed the effectiveness of all treatments (significant at *p* value < 0.01) to reduce the moisture content difference between soil layers. In case of temperature, only the treatment *Mixed* showed a clear positive effect in both *ME*_1_ and *ME*_2_ at increasing mean daily temperature values. The presented plot-specific results showed the significance of the treatments using *Plot* #1 as the level reference. The coefficients for the *ME*_2_ model showed how soil water dynamics were highly dependent on microsite conditions. All treatments were very highly significant (at least *p* value < 0.05) at increasing both moisture content and temperature while making moisture gradient more homogeneous from the 20 to 40 cm depth.

## 4. Discussion

The motivation of the experiment was to demonstrate the effectiveness of restoration techniques in arid and abandoned urban areas without soil preparation, and therefore the spatial variation of water-soil dynamics was inevitably part of the motivations of the study. The uncontrolled presence of coarse elements throughout the study area and the lack of soil preparation had an impact on how water infiltration behaved and how species responded to it, which is in line with previous research [18,35]. The experiment aimed at testing the robustness of the restoration techniques regardless of the suitability of the site for planting operations. Under those principles, soil texture homogenization and site preparation were not prescribed. As a result, the variation on soil conditions such as the presence of stones made the calibration process challenging and crucial due to the need to convert sensor measurements into variables of interest that decision makers can interpret. The conversion from sensor-based records into data for the modeling phase was the most-time consuming part of the data processing scheme. In this regard, the revealed differences between soil moisture content and temperature at both depths were similar. The installation of sensors at a fixed depth might be enough to describe water-soil dynamics. The increasing availability and widespread use of Synthetic Aperture Radar (SAR) could be a promising source of validation for further implementation of the presented methodology [36], which could potentially upscale the models to accurate wall-to-wall estimates using the large-scale coverage of remote sensing sources. Soil sensor measurements were effective at detecting microsite variability, a matter of concern when it comes to early-stage plantings on a small scale [18]. The spatial variation of soil conditions highly influenced the observed soil-water dynamics as previous studies in the field acknowledged [20,37]. In light of the results, the described calibration process can be improved if soil properties are as homogeneous as possible across the experiment. Therefore, soil preparation should always be a preliminary step not only to improve the effectiveness of restoration techniques, but also to reduce uncertainty when interpreting soil sensoring measurements and water-soil dynamics. Beside this, the three tested treatments increased moisture content and survival rate, more or less significantly, depending on the species and microsite properties.

The increment on the survival rate for the species *Pp* and *Al* when applying the treatments was minor compared to previous studies [38]. The survival rates for *Qi* or *Jt* were higher than expected as these species usually present problems when used in arid conditions [38]. However, the use of restoration techniques can significantly increase their survival rate [22]. Overall, the survival values for the *NoTreat* alternative are within the expected range [39], but the performance of treatments substantially differed when compared to other studies. For instance, the observed survival values were close to those reported by [27] using the same hydrogel product. However, the effectiveness of treatment *Mycorrhiza* in this study was slightly below the values reported in previous research [10,22,40]. The validation test (a sample of 30 individuals) revealed an inoculation rate of 22%. That underachievement of optimal inoculation rates has been previously reported [25,40], but in our study, the value was very low. Therefore, a better performance of solutions including mycorrhizas should be expected for the *Mycorrhiza* and *Mixed* treatments. The use of enhanced inoculation procedures [25,41] might overcome this limitation observed after planting.

The analyses on soil volumetric water content showed, in line with other studies [15], the benefit of restoration treatments. Even with the observed low increment in terms of soil volumetric content, the survival rate increased up to 10% and the infiltration of water became more tedious after rainfall episodes, leading to increase water availability and the avoidance of runoff episodes. In the Mediterranean region, where water is by far the most limiting resource for plant growth, minimizing water runoff is crucial for tree species survival [42]. Therefore, efforts in green urban planning when it comes to creating new urban forests should be focused on early-stage soil preparation [11] as even in the absence of watering systems, plants have a better chance for survival. The results also show the increasing effects treatment had on soil temperature, which can be expected if soil moisture content also increases. Both phenomena explain the enhanced growing conditions because of the restoration techniques.

**Figure A1:**
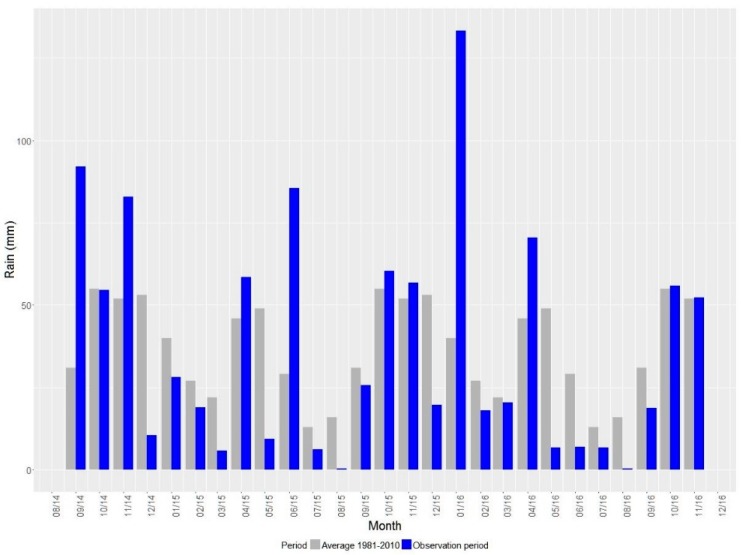
Monthly distribution of precipitation in the study area during the period 1981–2010 and during the experiment lifecycle.

It is important to emphasize that weather conditions in the city of Valladolid during the experiment were very challenging, particularly regarding the distribution of precipitations with high variation of monthly rainfalls (very dry summers and heavy isolated rainfalls) compared to the historic data (Figure A1). The spatial variation of soil conditions and its implications need to be assessed in new urban forests projects. The effectiveness of treatments at improving the survival rate of tree species was already known, but their contribution to minimize water run-off was clearly demonstrated in this study. Based on these results and on existing literature [9], resources in green urban planning should be devoted to improving soil preparation not only to an adequate soil texture but also to create soil physiological uniformity. The investment in soil preparation can be reduced by testing resource-friendly solutions such as compost amendments that can increase economic opportunities for local compost producers [11,43], which makes sense within the peri-urban environment.

The application of the presented methodology in more controlled conditions would favor a better understanding of soil-water dynamics and reduce the level of complexity of the calibration phase, a must when using soil sensoring methodologies [44]. In this study, the application of restoration techniques was systematic and not based on soil sensors information. In fact, the presented approach could be implemented and evaluated before planting operations, with the aim of mapping areas of different water availability and pre-designing the application of restoration techniques based on that information.

## 5. Conclusions

Green urban planning and land managers have the opportunity to optimize the creation of new urban forest areas by incorporating innovative measuring systems capable of integrating multiple sources of data. In this perspective, the study represents a novel big data solution that combines detected information from soil sensors, atmospheric data, and field observations that, together, are used to monitor and evaluate the establishment of a new urban forest. The use of soil sensors highlighted spatial patterns in soil-water dynamics and the tested restoration techniques contributed to increased water retention and tree survival despite the challenging conditions of the study area and the experiment.

## Figures and Tables

**Table 1:** Mean values, standard deviations (in brackets), and coefficients of significance for models ME_1_ and ME_2_.

Factors	ME_1_				ME_2_			
	Log (*Hum*_20_)	*Hum*_20_–*Hum*_40_	*Tem*_20_	*Tem*_40_	Log (*Hum*_2__0_)	*Hum*_20_–*Hum*_40_	*Tem*_20_	*Tem*_40_
*Mycorrhiza*	0.099	−3.651	0.094	0.048	0.099	−3.850	0.095	0.048
	(0.060)	(2.072)	(0.196)	(0.184)	(0.048)	(1.398)	(0.160)	(0.152)
	*	*				***		
*Retainer*	0.051	−3.601	0.270	0.265	0.051	−3.800	0.271	0.266
	(0.071)	(1.648)	(0.250)	(0.260)	(0.048)	(1.398)	(0.160)	(0.152)
		**				***	*	*
*Mixed*	0.027	−4.167	0.504	0.497	0.027	−4.366	0.504	0.498
	(0.083)	(2.180)	(0.198)	(0.185)	(0.048)	(1.398)	(0.160)	(0.152)
		*	**	***		***	***	***
*Plot* #2					0.187	−7.211	−1.162	−1.096
					(0.048)	(1.374)	(0.160)	(0.152)
					***	***	***	***
*Plot* #3					0.168	−8.317	−1.257	−1.130
					(0.048)	(1.374)	(0.159)	(0.151)
					***	***	***	***
*Plot* #4					0.096	−6.209	−0.366	−0.368
					(0.048)	(1.398)	(0.160)	(0.152)
					**	***	**	**

**Figure A2:**
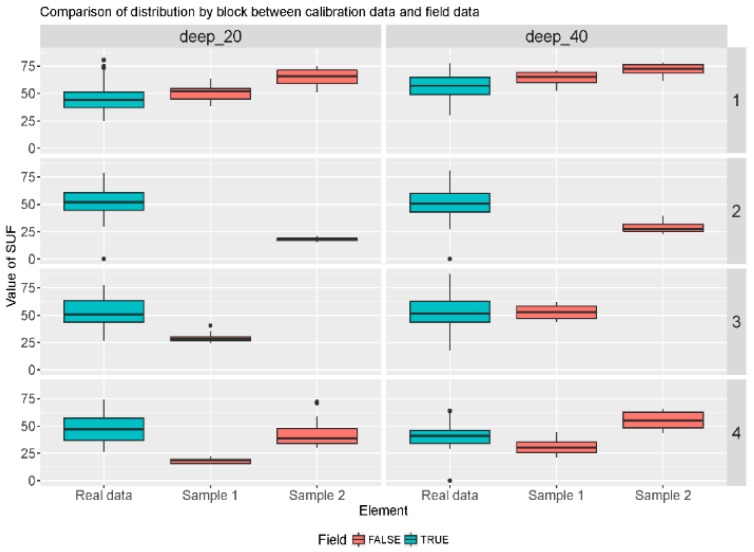
Mean value of frequency units of the capacitance (SUF) computed in the sampling plots.

**Figure A3:**
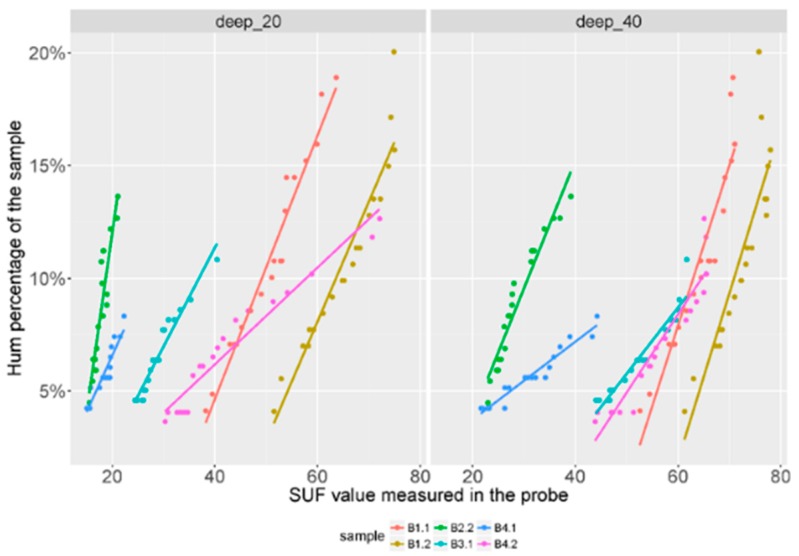
Mean value of frequency units of the capacitance (SUF) in the samples used for calibration measured in the field and in the laboratory before starting the drying process (above). Final models used to calibrate sensor measurements for each sample (below).

## Appendix A

**Table A1:** Texture and soil profile characteristics.

Sample	Block	Depth	Sand (%)	Silt (%)	Clay (%)	Texture
A	1	0–37 cm	57	27	16	Loam-Sandy
	2	0–20 cm	64	18	18	Loam-Sandy
	3	0–20 cm	66	16	18	Loam-Sandy
	4	0–30 cm	43	30	27	Loam
B	1	37–80cm	63	17	20	Loam-Sandy
	2	20–50 cm	73	16	1	Loam-Sandy
	3	20–50 cm	77.5	21	1.5	Loam-Sandy
	4	30–90 cm	43	25	32	Loam
C	2	50–120cm	96	3	1	Sandy
	3	50–130 cm	97	2	1	Sandy

**Table A2:** List of the tree species and their corresponding Mycorrhiza species.

Host Tree Species	Mycorrhiza Species
*Juniperus thurifera*	*Rhizophagus* spp. (Endo).
*Prunus dulcis*	*Rhizophagus* spp. (Endo).
*Acer campestre*	*Rhizophagus* spp. (Endo).
*Pinus pinea*	*Pisolithus tinctorius* (Pers.) Coker & Couch (Ecto).
*Quercus ilex*	Pisolithus + *Scleroderma polyrrhizum* (JF Gmel.) Pers (Ecto).

## Appendix B

Soil sensed measurements needed to be converted into operational units. Sensor measurements showed alternative soil-dynamics patterns across the study area. The observed trends considerably varied both spatially and between soil layers (Figure A2). Frequency capacitance records were converted into soil volumetric water, and then into moisture content (expressed as %) through a calibration procedure based on soil samples. In our study, we collected two soil samples in each of the four sampling plots, and then these measurements were used to calculate soil volumetric water. The sites selected for the sampling were representative when compared to the measured mean SUF value with the sensors. The process was as follows: soil samples were weighed several times in the laboratory before and after drying conditions. Soil sensor records were also collected before and after drying conditions. As a result, we modelled for each sampling plot the relationship between real soil volumetric content and sensor information along a gradient of moisture intervals (Figure A3). Once the model relationships were built, sensor measurements before and after the calibration test were converted and integrated into the data processing system.
